# A skin explant model for studying UV‐induced DNA damage and repair

**DOI:** 10.1111/php.14070

**Published:** 2025-02-03

**Authors:** Hailey Payne, Christina Athans, Shiyong Wu, Veronica Bahamondes Lorca

**Affiliations:** ^1^ Honors Tutorial College Chemistry Ohio University Athens Ohio USA; ^2^ Honors Tutorial College Biological Sciences Ohio University Athens Ohio USA; ^3^ Chemistry and Biochemistry Department Ohio University Athens Ohio USA; ^4^ Edison Biotechnology Institute Ohio University Athens Ohio USA; ^5^ Departamento de Tecnología Médica, Facultad de Medicina Universidad de Chile Santiago Chile; ^6^ Present address: HCOM Ohio University Athens Ohio USA

**Keywords:** DNA damage and repair, mouse skin explant model, skin, solar ultraviolet

## Abstract

There is a growing need for a skin model that combines the natural physiology of skin while reducing reliance on mice. Natural physiology is achieved by using fresh, intact skin explants sourced from living organisms such as humans or mice. This study focused on the standardization and characterization of an in vitro mouse skin explant model for investigating solar ultraviolet (sUV)‐induced skin damage. We developed a protocol to use skin explants derived from the discarded tissue of mice after euthanasia. These explants consist of intact dermal and epidermal layers suspended in cell culture medium and maintained in vitro. To assess the viability of the skin explants, we evaluated tissue morphology (via hematoxylin and eosin [H&E] staining), viability markers, and DNA damage markers. Our ex vivo model preserves the key characteristics and physiological responses of in vivo skin for short incubation periods, while minimizing the use of mice. This model enables the study of DNA damage and repair, and it has broad applications, including studies on skin photoprotection, topical treatments, drug development, and cosmetics.

## Graphical abstract

The study describes an ex vivo skin model that preserves the natural physiology of the skin while addressing ethical concerns related to animal research. We demonstrate this model maintains key characteristics and physiological responses of in vivo skin during short incubation periods. Moreover, this model requires fewer mice than in vivo testing, thus reducing research costs by enabling the study of multiple variables using skin from a single mouse.

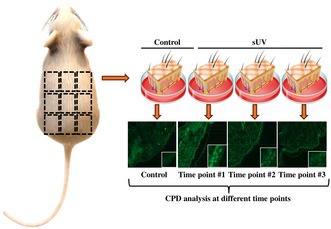

**Abbreviations**
CPDs: cyclobutane pyrimidine dimers
DMEM: Dulbecco’s Modified Eagle Medium
FBS: fetal bovine serum
GAPDH: Glyceraldehyde 3‐phosphate dehydrogenase
H&E: Hematoxylin and eosin
sUV: solar ultraviolet
γ‐H2AX: gamma‐H2AX

## INTRODUCTION

An ideal skin model should maintain the natural physiology of skin and avoid ethical issues in research. Additionally, this model should include the various cell types and cellular organizations that characterize the skin. Natural physiology is studied using fresh, intact skin explants sourced from living organisms, such as humans or mice. However, using skin from living donors raises ethical concerns, as it involves the use of animals in research. 2D cell culture is a commonly used method for studying skin, but it has limitations, typically lacking the diversity of cell types and the cell shape and morphology of intact skin. An alternative approach is to use 3D (reconstructed skin) models to study skin.^1^, ^2^ While these models address ethical concerns, they still do not replicate all the physiological structures found in living tissue, such as melanocytes, immune cells, and blood vessels, and they can be expensive to use and maintain. Instead, skin explants—pieces of intact dermal and epidermal tissue—are cultured in cell culture medium and maintained in vitro, preserving more of the natural structure and function of the skin.^3^

Current literature on ex vivo human skin explants is limited and presents a variety of results and methods for enhancing skin preservation.^4^, ^5^, ^6^, ^7^ The organ culture of human skin has a long history of development.^8^ Generally, human skin samples, donated from reconstructive surgeries, are sterilized, cut into pieces, with subcutaneous fat removed, and incubated in an air‐liquid interface, with the dermis submerged in culture medium.^3^, ^5^, ^6^, ^7^ Frade et al. (2015) tested their Human Organotypic Skin Explant Culture model for up to 75 days. Human donor tissue was cultured under conditions using Dulbecco's Modified Eagle Medium (DMEM), fetal bovine serum (FBS), antibiotics (penicillin, streptomycin, and amphotericin B), and L‐glutamine on a metallic grid scaffold. Histological analysis showed that the tissue appeared completely normal on Day 7. By Day 30, the epidermis had a reduced stratum spinosum, though the overall cutaneous structure remained unchanged. By Day 75, the skin had thinned, but the dermal‐epidermal junction appeared intact, suggesting the tissue was still similar to normal skin at this point.^7^ In contrast, Neil et al. (2020) found that their human skin explant model remained viable for up to 9 days, after which necrosis, parakeratosis, and spongiosis began to occur.^9^ In their study, a different culture medium was used, containing DMEM and Ham's F12, supplemented with fetal calf serum, antibiotics (penicillin, streptomycin, and amphotericin B), hydrocortisone, adenine, insulin, epidermal growth factor, and triiodothyronine (T3).^9^, ^10^ These studies clearly show that no single protocol has been standardized for the use of human skin explants, and it remains uncertain which protocol is best for the long‐term viability of skin explants. Furthermore, the utility of this model is constrained by the limited availability of surgical donors, which restricts its widespread application.

While human skin explants offer valuable insights, using mouse tissue can help validate the explant results against in vivo testing. However, data on mouse skin explants are even more limited. One key advantage of using the skin explant model is that it requires fewer mice compared to in vivo testing for, for example, studies of skin DNA damage or skin photoprotection. In traditional in vivo testing, one mouse is used per time increment, meaning several mice are needed for each experiment. In contrast, in vitro testing with ex vivo skin explants allows a single mouse to provide information on multiple time points or treatments, thereby reducing the ethical concerns associated with animal use. This approach not only minimizes the number of animals required but also yields more reliable data, as it eliminates the variability introduced by different mice populations. These advantages make the ex vivo skin explant model a valuable tool for studying a wide variety of applications such as topical treatments and skin biology.

## MATERIALS AND METHODS

### Skin collection and maintenance

The skin of healthy wild‐type SKH1 mice was used in this study. Skin was collected from mice that, due to other procedures, needed to be euthanized. After sterilization with 70% ethanol, the skin was first removed from the backs of the mice. Next, the fat and connective tissue were separated from the skin so only the dermis and epidermis remained. Since the detached skin curls easily, it was placed on a synthetic scaffold made of polycaprolactone developed previously in the laboratory.^11^ The skin‐scaffold piece was placed on a mixture of cell culture medium with the dermis inside and the dry epidermis outside of the medium. Two different medium solutions were compared, described by others^4^, ^7^, ^9^, ^10^ to keep explants viable for the longest duration of time (Table 1). The explants were maintained in an incubator at 37°C and 5% CO_2_ until collection for analysis. The medium was changed every 2 days.

**TABLE 1:** Medium mixtures.

Medium 1	Medium 2
DMEM (Corning®, 10‐013‐CV) 10% Fetal Bovine Serum (R&D Systems) 1% penicillin/streptomycin (Corning®, 30009CI) 2 mM L‐Glutamine (Corning®, 30009CI)	DMEM/F12 (Gibco™, 12,634,010) 10% Fetal Bovine Serum (R&D Systems) 1% penicillin/streptomycin (Corning®, 30009CI) 2 mM l‐Glutamine (Corning®, 30009CI) 1 μg/mL (−)‐Isoprenaline hydrochloride (Sigma‐Aldrich®, I6504) 10 ng/mL Epidermal Growth Factor (Stemcell Technologies™, 78016) 1.4 ng/mL T3 (Stemcell Technologies™, 100‐0548) 0.4 ng/mL Hydrocortisone (Stemcell Technologies™)

### Analysis of frozen tissue explants

The skin explants were prepared as previously described, but immediately after cutting the tissue, some were placed into tubes and frozen at −80°C with or without the polycaprolactone scaffold for 10, 20, or 30 days. For analysis, the tubes containing the frozen samples were taken out of the −80°C freezer and placed on ice to thaw for 10 min. Next, the tubes were allowed to thaw to room temperature for 10 min. Finally, the tissues were removed from the tubes and placed on the polycaprolactone scaffold in medium and rested for 5 min before further treatment.

#### Hematoxylin and eosin (H&E) staining and histological evaluation

For the analysis, tissues were fixed in 10% v/v buffered formalin for 24 h at room temperature and then rinsed for 1 h in running water. Samples were stored at 4°C in 70% ethanol until embedding. The tissues were embedded in paraffin and sectioned (4 μm thickness) for histology (H&E) and immunohistochemistry analysis.

### Protein extraction and western blot

A portion of the samples was also frozen and used for protein extraction. A small portion (~2 mm^2^) of the frozen tissue was first placed into a mortar and submerged in liquid nitrogen until it froze and then rapidly crushed with a pestle and ground into a paste. Next, a mixture of Radioimmunoprecipitation assay (RIPA) buffer (100 mM Tris–HCl, 2% v/v Triton X‐100, 300 mM NaCl, 0.2% w/v SDS, 10 mM EDTA, and 1% w/v sodium deoxycholate) with the phosphatase and protease inhibitors, 1× PhosSTOP (Roche, 4906845001) and 1X Complete Mini, EDTA‐free Protease Inhibitor Cocktail (Roche, 11836170001) respectively, was added to the paste and further crushed into a liquid. The liquid was transferred to a smaller glass mortar and pestle over ice to be ground further. The liquid was transferred to a tube and sonicated (Sonic Dismembrator 550, Fisher Scientific, F1996) on power 1.5 for 5 s 3 times. The antibodies GAPDH (1:2000, Santa Cruz Biotechnology, sc‐365,062), β‐actin (1:1000, Santa Cruz Biotechnology, sc‐47778), and γ‐H2AX (1:1000, Cell Signaling, 9718) were used.

### Immunostaining of cyclobutane pyrimidine dimers (CPDs)

Tissue sections (4 μm) were dewaxed and rehydrated using xylol (3 times of 10, 5, and 5 min each) then 100% (3 times of 10, 5, and 5 min each), 95% (5 min), 70% ethanol (5 min), and water (5 min). Then, the antigens were recovered by heat (0.01 M citric acid, pH 6.0) for 10 min at 92°C. Next, the DNA was denatured using 0.2 mM NaOH in 70% ethanol for 30 min. Samples were blocked using 20% fetal bovine serum in 1× PBS for 30 min. Finally, tissues were incubated overnight at 4°C with the primary antibody anti‐Cyclobutane Pyrimidine Dimers (CPDs) [Clone: TDM2] (1:1500, Cosmo Bio Co. Ltd.) in a 5% fetal bovine serum in 1X PBS. After washing with 1X PBS, cells were incubated with the corresponding secondary antibodies. Pictures were taken using the inverted microscope Zeiss Axio Observer 5.

### sUV irradiation of tissue explants

The skin was irradiated with a single dose of sUV, using 180 mJ/cm^2^ of UVB from a UVA/UVB solar lamp (UVA‐340 lamp, Q‐Lab Corporation) previously characterized.^12^ After irradiation, the samples were incubated (37°C and 5% CO_2_) for the specified periods of time.

### Isolation of primary fibroblast

After the mice died naturally or were euthanized due to IACUC requirements, skin samples were extracted from the mice following a protocol adapted from reference.^13^ To extract the skin, the mice were first sterilized in 70% ethanol alcohol for at least 15 min. The dorsal skin of the mice was dissected, avoiding the limbs and any large fat deposits typically located in the abdominal and lateral abdominal regions of the mice. The skin samples were cut into roughly 1.5 cm^2^ pieces, and they were first rinsed with a solution of 1× PBS and Gentamicin (50 ng/mL, Gibco, 15750‐060). Next, the skin was treated with a cold 0.5% dispase solution (5 U/mL dispase in Hanks' Balanced Salt Solution, #07913, Stemcell™) in DMEM medium (Corning™, Cell gro™, 10‐013‐CV) and incubated at 37°C for 45 min. After rinsing with PBS, the skin layers were separated, and the epidermis was discarded. The dermis was incubated in a 15 mL tube with 10 mL of collagenase type IV (1 mg/mL, Gibco, 17104‐019) in DMEM to allow the isolation of individual cells. After centrifuging and collection of the pellet, the cells were seeded and grown in DMEM medium (Corning™, Cell gro™, 10‐013‐CV) supplemented with 10% v/v FBS and 1% v/v penicillin/streptomycin at 37°C with 5% CO_2_. Primocyn™ (100 μg/mL, Invivogen, #ant‐pm‐05) and Fungin™ (10 μg/mL, Invivogen, ant‐fn‐1) were added to the cell culture medium and maintained for 5 days to prevent microbial and fungal contamination.

### DNA extraction and ELISA

DNA extraction was performed using the DNeasy Blood and Tissue Kit (Qiagen, 69504), following the manufacturer's indications. The formation of CPDs in the DNA samples was detected and quantified using the OxiSelect™ UV‐Induced DNA Damage ELISA Kit (Cell Biolabs Inc., STA‐322), following the manufacturer's protocol.

## RESULTS AND DISCUSSION

### Skin explants show similar characteristics to fresh mice skin tissue

**FIGURE 1:**
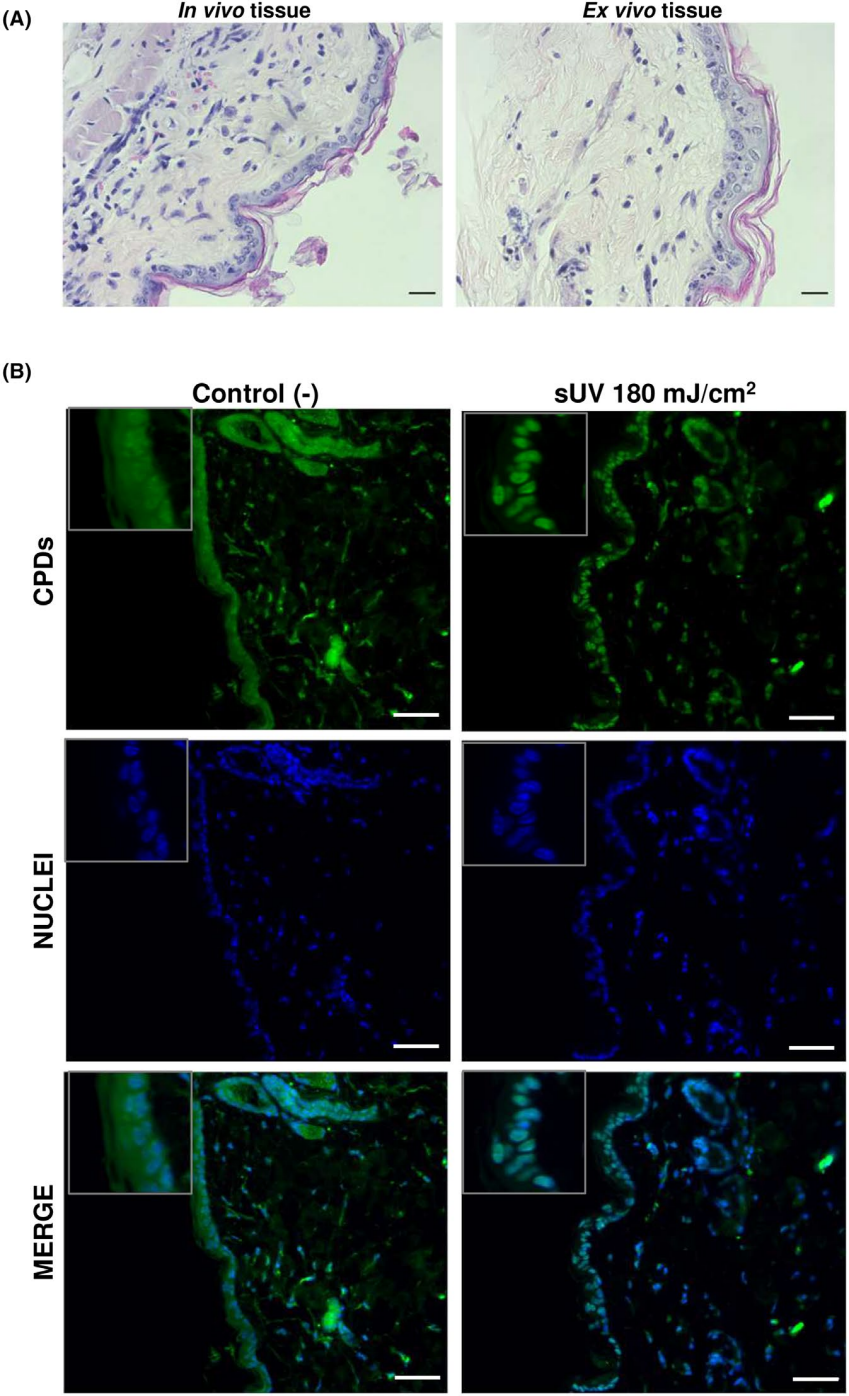
Skin explants show similar characteristics to fresh mice skin tissue. (A) Representative histology of in vivo mice tissue and ex vivo explant tissue that was incubated for 24 h in DMEM medium *N* > 2. Samples were stained with H&E. Bar 20 μm. (B) Representative immunofluorescence of CPD staining on sham and sUV‐irradiated explants after 180 mJ/cm^2^ (UVA‐340 lamp, Q‐Lab Corporation). CPDs are shown by the expression of bright green nuclei. Nuclei are stained blue in the middle section. Merge is the conjoining of the CPD and nuclei images. Bar 50 μm. *N* > 2.

To evaluate the maintenance and viability of tissue after removal from mice, the morphology of skin samples from an in vivo mouse and a 24‐h ex vivo mouse explant was compared using H&E staining. Figure 1A shows the histology of both an in vivo and an ex vivo mouse tissue sample incubated for 24 h in DMEM. Both samples were embedded in paraffin and stained with H&E. The results in Figure 1 indicate that both epidermises contain an approximately equal number of stained nuclei with normal morphology, suggesting that both tissues have similar quantities of viable cells within the epidermis. Additionally, it is commonly observed that the dermal–epidermal junction is separated in skin explants. However, as shown in Figure 1A, no gaps are present between the dermis and epidermis in the explant tissue after 24 h of incubation and are similar to that of in vivo tissue. These findings suggest that the morphology of ex vivo explant tissue is well‐preserved for at least 24 h compared to in vivo tissue.

To assess the response of skin explants to solar UV (sUV) exposure and the ability to observe the induction of DNA damage, we evaluated the levels of CPDs on the skin explants by immunostaining. CPDs are a very common form of sUV‐induced DNA damage, so measuring the rate of formation is a useful tool for determining if/when the tissue is still responding and is viable. Figure 1B shows the levels of CPDs on sham samples (without sUV exposure) and sUV‐irradiated samples with 180 mJ/cm^2^ of UVB from our sUV source.^12^ As shown in Figure 1B, nuclear staining with DAPI confirms that both sham and sUV‐treated tissue explants have an approximately equal number of epidermal nuclei (stained blue), indicating a similar number of cells and cell layers. For the CPD signal, sham‐irradiated samples have a dull green background in the epidermis, with no nuclear staining, while the sUV‐treated samples show bright green staining of the nuclei in the epidermal cells (Figure 1B). These findings suggest that CPDs are a reliable marker for assessing sUV‐induced damage and that CPDs are formed following sUV exposure in our ex vivo tissue explants.

### Long‐term explants show minimal morphological changes up to Day 6 and were unable to preserve their viability for more than 13 days

**FIGURE 2:**
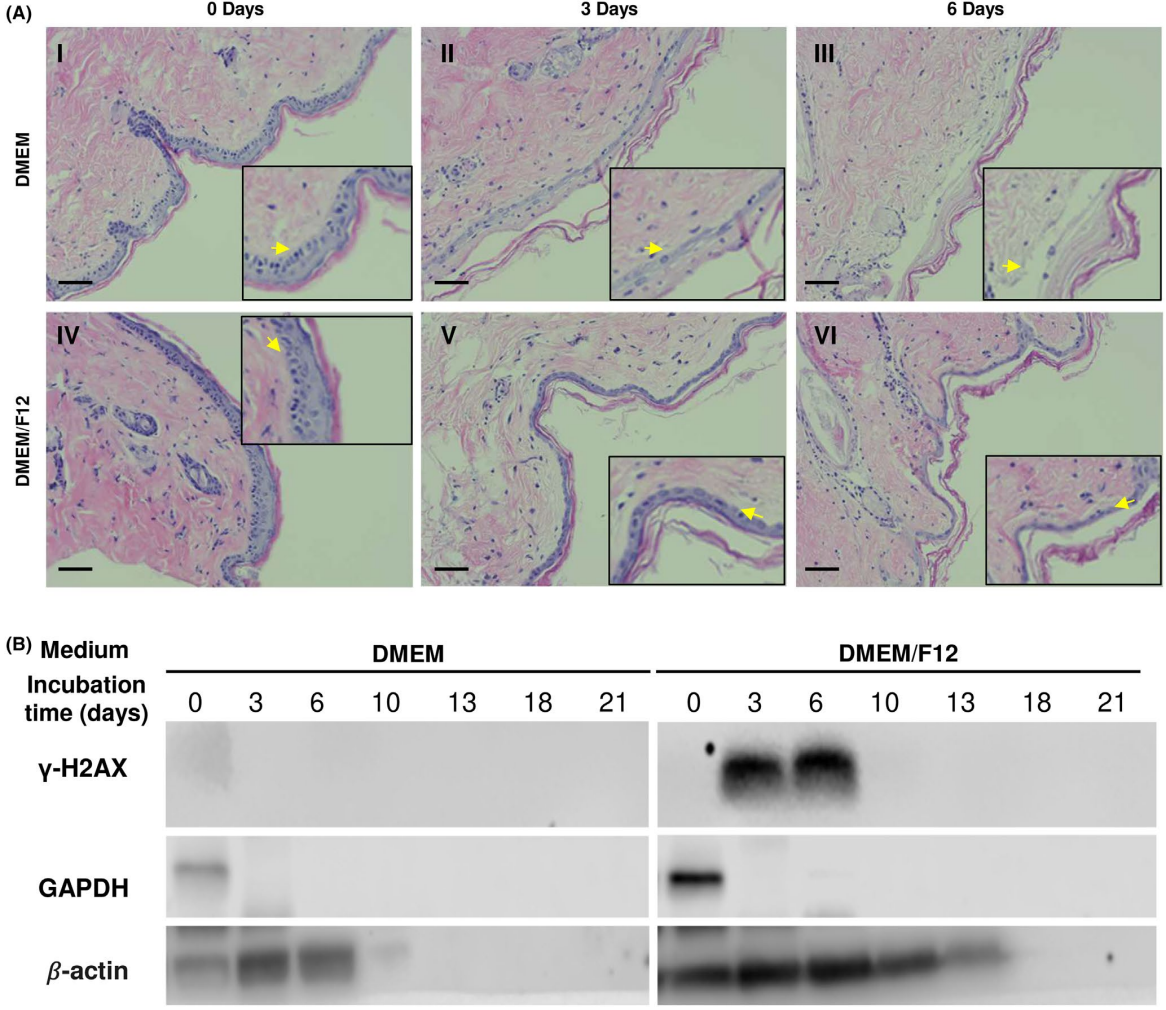
Histology and viability of long‐term explants. (A) H&E‐stained ex vivo tissue incubated for up to 6 days after extraction. Tissue explants were incubated in DMEM (I–III) and DMEM/F12 (IV–VI). The yellow arrow in the inserts shows the dermal–epidermal junction. Bar 50 μm. (B) Viability of long‐term ex vivo explants maintained for up to 21 days. Tissue explant samples were incubated in DMEM or DMEM/F12 media mixtures. The housekeeping proteins GAPDH (conserved on Day 0) and β‐actin (conserved until ay 10), and the DNA damage marker γ‐H2AX were evaluated via Western blot. *N* = 2.

To assess the preservation of ex vivo explants at different incubation times, mouse tissues were placed in designated culture media (Table 1) and harvested at 0, 3, 6, 10, 13, 18, 21, 25, and 28 days post‐extraction. Histological analysis was performed to evaluate the characteristics of the nuclei and the status of the epidermal–dermal junction. Figure 2A shows representative H&E staining of samples cultured in DMEM (medium 1) and DMEM/F12 (medium 2) mixtures (as detailed in Table 1). Figure 2A‐II and V show that by Day 3, the epidermis of the explants maintained in both medium is thinner compared to the zero‐day control explant. Additionally, and as shown by Figure 2A‐VI, tissues cultured in the DMEM/F12 mixture maintain the most intact dermal–epidermal junction (indicated by the yellow arrow) up to Day 6. However, by Day 10, all samples exhibit a disconnected dermal–epidermal junction (not shown). This result suggests that the incorporation of supplementation (hormones, growth factors, minerals, etc.) helps to achieve better longevity of the explants.

In addition to H&E staining, the viability of long‐term skin explants was assessed by evaluating the expression of the DNA‐damage repair protein gamma‐H2AX (γ‐H2AX), as well as the housekeeping proteins β‐actin and glyceraldehyde 3‐phosphate dehydrogenase (GAPDH). GAPDH, a key enzyme in glycolysis, and β‐actin, a structural protein, both exhibit decreased levels with age and improper tissue preservation.^14^ The tissues, incubated in their respective medium mixtures, were harvested at 0, 3, 6, 10, 13, 18, 21, 25, and 28 days after extraction. Figure 2B shows the protein expression levels of γ‐H2AX, β‐actin, and GAPDH up to 21 days for the different medium mixtures listed in Table 1. The results indicate that both mediums maintain GAPDH expression only at the first time point, while β‐actin is detectable up to Day 10 for DMEM and up to Day 13 for the DMEM/F12 mixture. The rapid decline in GAPDH levels versus to β‐actin, could be attributed to GAPDH's role as a functional marker, making its expression sensitive to cellular stress, whereas β‐actin is more stable for being a structural protein. Notably, the DNA damage marker γ‐H2AX was observed in samples incubated with the DMEM/F12 mixture, suggesting ongoing DNA damage/stress.

### Frozen tissue explants preserve their viability and capability to respond to sUV

**FIGURE 3:**
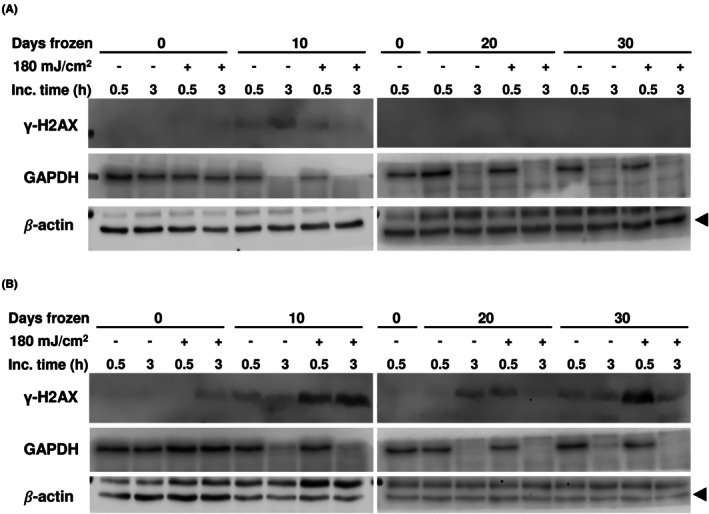
Viability of frozen explants and capability to respond to sUV. Explants were extracted from two male (A) and two female (B) mice and frozen for 0, 10, 20, and 30 days. Explants were exposed to sUV, and protein extracts were analyzed at 0.5 and 3 h. GAPDH is conserved until Day 30 on 0.5‐h incubations, β‐actin is conserved until Day 30, and γ‐H2AX shows minimal expression in males and more expression in females with increasing time. *N* = 2. Arrowheads indicate β‐actin band.

Since the morphology of fresh explants in culture medium was only maintained for up to Day 6, the viability and capability to respond to sUV of frozen explants were determined. Skin explants collected from discarded tissues of two male and two female mice immediately after euthanasia were extracted and, except for the Day 0 sample, frozen immediately and maintained at −80°C for 10, 20, and 30 days. For analyzing the viability and the capability to respond to sUV, samples were thawed and placed in the DMEM/F12 mixture given that the morphology, dermal epidermal junction (Figure 2A), and β‐actin expression (Figure 2B) were most conserved in this medium. Then, these samples were sham‐ or sUV‐irradiated and incubated for 0.5 or 3 h. The same procedure was applied to the Day 0 samples, which were used as control. Figure 3A versus 3B show little difference between males and females; however, for females, the γ‐H2AX expression was higher than in males. Notably, starting on Day 10, the GAPDH expression decreased for both sham‐ and sUV‐exposed samples incubated for 3 h after defrosting and irradiation. The GAPDH trend continued for the 20‐ and 30‐day frozen tissues for both males and females. Additionally, there is little change in β‐actin expression from 0 to 30 days frozen for both males and females (Figure 3A vs. 3B). These results indicate that GAPDH is only present at time periods shorter than 3 h in the tissues frozen for at least 30 days. On the other hand, β‐actin is expressed evenly throughout the 0–30 days frozen for both males and females (Figure 3A,B) indicating that the tissue can be frozen for at least 30 days. The quick lack of GAPDH could be due to the fact that this protein is a functional marker compared to β‐actin, which is a structural protein. Therefore, we assume that the viability of the tissue explant is altered at a 3‐h incubation time, but at short times, we can still trust the responsiveness of this model. Finally, γ‐H2AX was only expressed in the female mice at seemingly random times (Figure 3B). This might suggest that the tissue is experiencing DNA stress from the freezing and thawing process. However, it seems to respond to sUV at 30 min, a result that agrees with the idea that the viability and responsiveness of our skin explant model are conserved at short periods of time, independently of whether the tissue was frozen.

**FIGURE 4:**
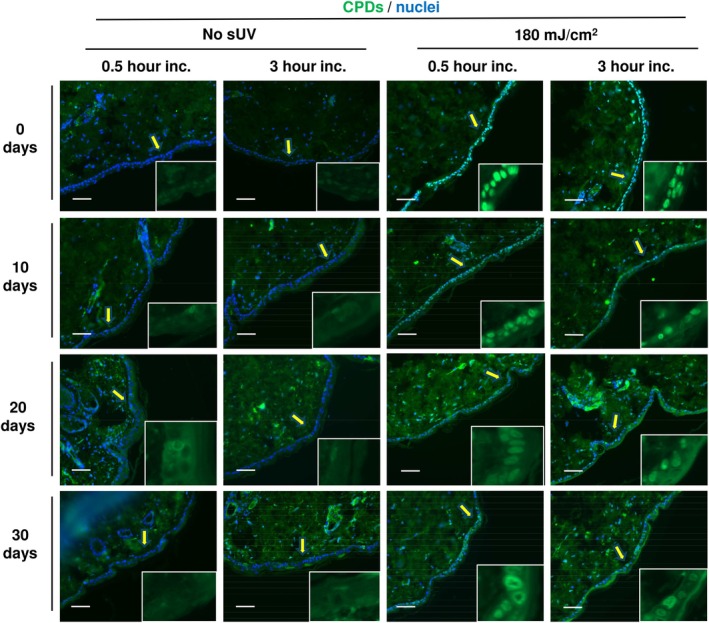
Capability of frozen explant to form cyclobutane pyrimidine dimers (CPDs). Images show CPD formation on explants from two male mice frozen for 0, 10, 20, and 30 days. CPD (green) and nuclei (DAPI, blue). Yellow arrows indicate the site shown by the insert. Insert only shows CPD signal. Bar 50 μm.

**FIGURE 5:**
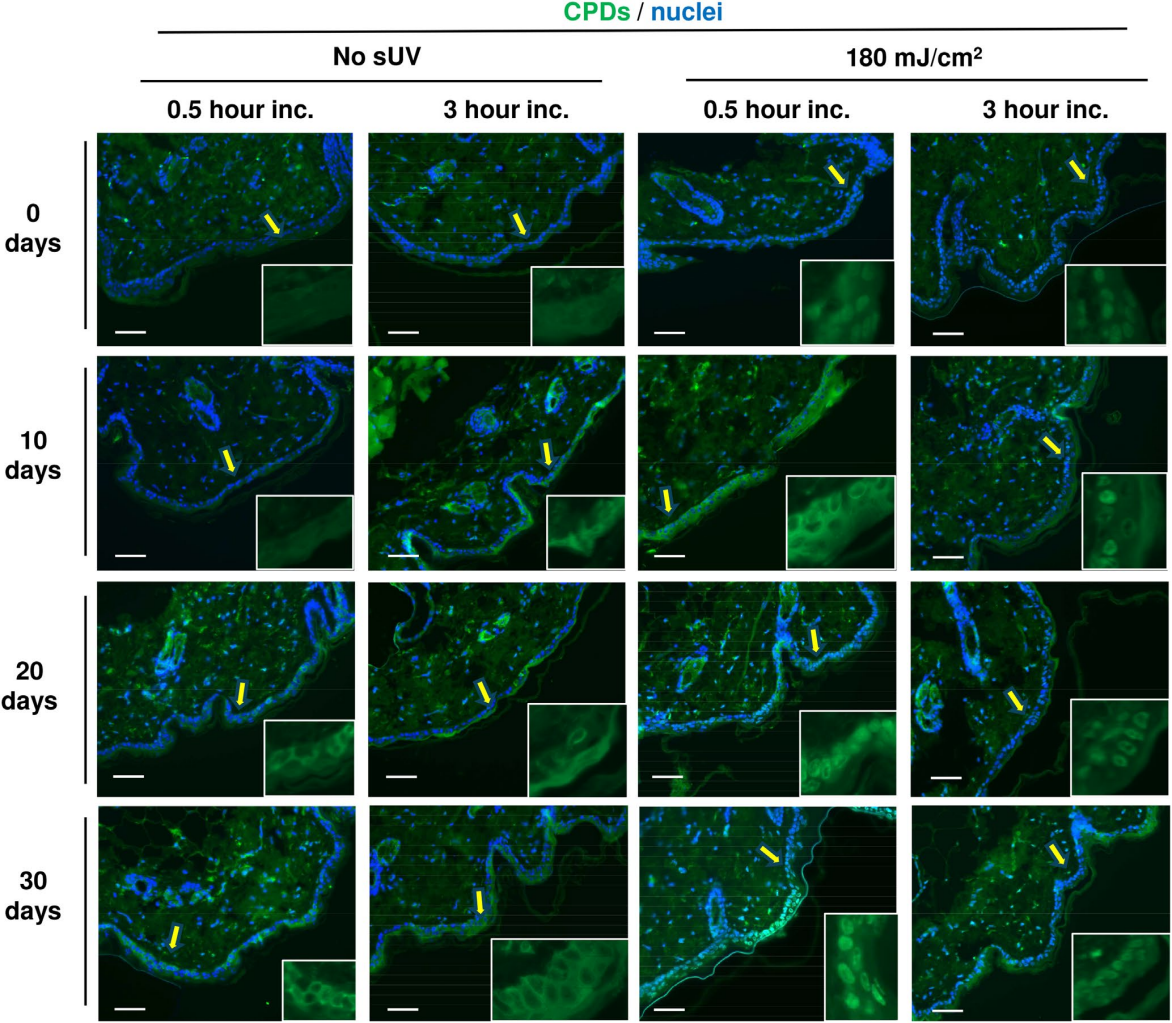
Capability of frozen explant to form cyclobutane pyrimidine dimers (CPDs). Images show CPD formation on explants from two female mice frozen for 0, 10, 20, and 30 days. CPD (green) and nuclei (DAPI, blue). Yellow arrows indicate the site shown by the insert. The insert only shows CPDs signal. Bar 50 μm.

To further analyze the capability to respond to sUV of the frozen explants, their ability to form CPDs after thawing and exposure to sUV was evaluated. Figures 4 and 5 show the CPD formation in the ex vivo explants of male (Figure 4) and female (Figure 5) after thawing, sUV irradiation, and incubation for 0.5 or 3 h. For males (Figure 4) there seems to be little difference in CPD formation between 0.5 and 3 h post‐sUV, whereas for females (Figure 5), the CPD formation is highest 0.5 h post‐irradiation and decreases during the 3 h incubation period. These sets of samples demonstrate that CPD formation is absent in the non‐irradiated samples and present in the sUV‐exposed samples, even when they were previously frozen.

In this experiment, we chose to freeze the skin explants immediately at −80°C, as this was the simplest method for preserving tissue viability. This approach did not significantly impact our experimental outcomes, as our study focused on short‐term tissue viability, particularly immediately after thawing for explant cultures. While the use of a cryopreservation solution and controlled‐rate freezing is important for long‐term tissue preservation,^15^ these methods were not necessary for the goals of our study.

### Skin explants successfully reproduce CPD formation and repair results from primary cells

**FIGURE 6:**
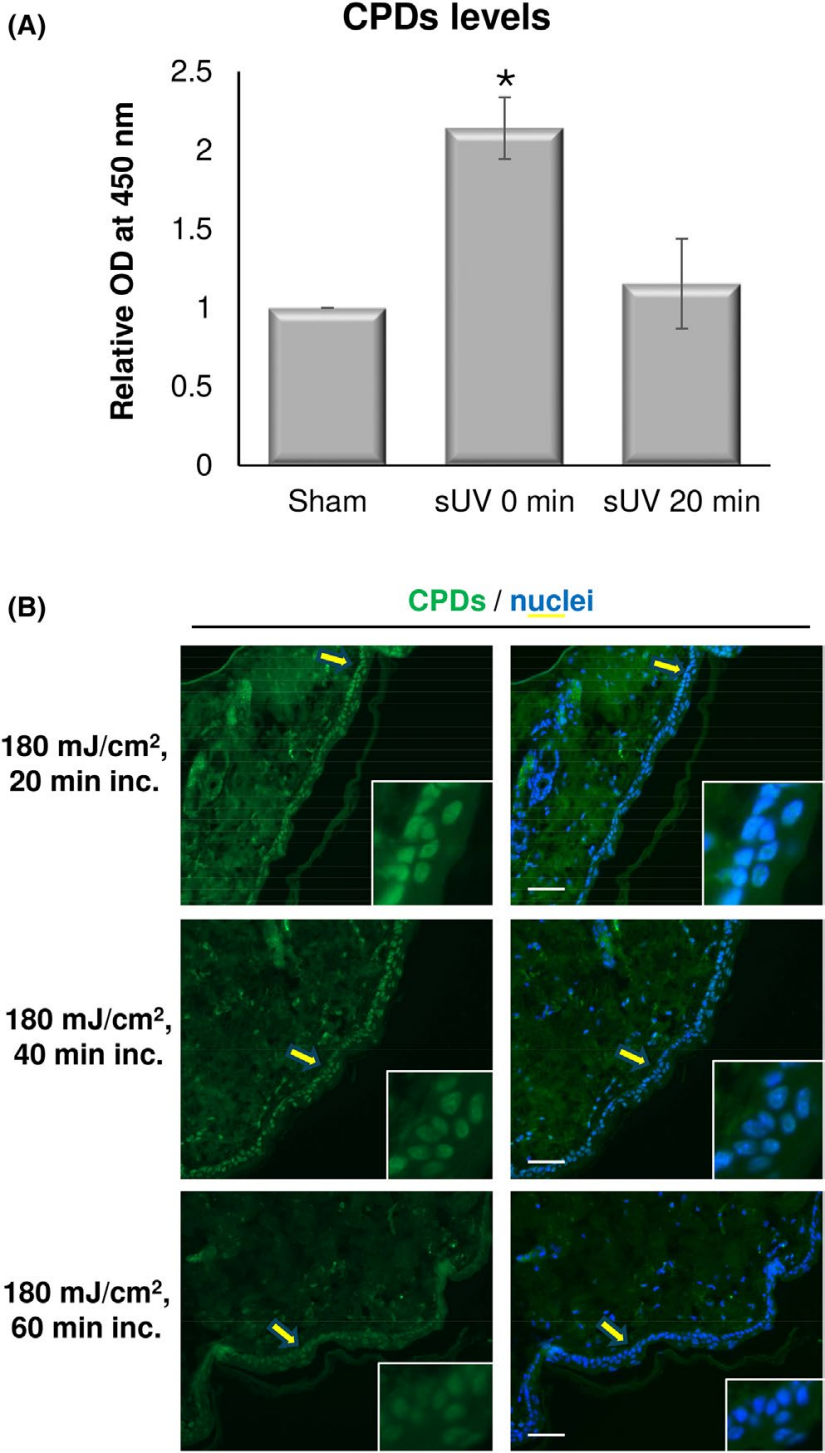
CPDs repair capacity on tissue explants versus primary cells. (A) Bar plot showing the quantified levels of CPDs detected in primary skin fibroblasts isolated from a male wild‐type SKH1 mouse. The plot shows a quick increase in CPDs immediately after sUV, which then decreased after 20 min of incubation. *N* = 3. (B) Pictures show CPD formation in skin explants collected from a wild‐type mouse at 20, 40, and 60 min post‐sUV irradiation. *N* = 1. Yellow arrows indicate the site shown by the insert. CPD (green) and nuclei (DAPI, blue). Bar 50 μm. *p < 0.05 was considered significant.

In our research laboratory, we were working in parallel analyzing the formation of CPDs in primary fibroblasts isolated from male and female SKH1 mice. Fibroblasts from a wild‐type male SKH1 mouse were either sham‐ or sUV‐irradiated, and the levels of CPDs were measured via ELISA at early time points post‐sUV to capture both CPD formation and the initial repair process. As shown in Figure 6, in our primary cell line, CPDs increased immediately after sUV irradiation and were quickly repaired since CPDs' levels significantly decreased 20 min post‐sUV exposure (Figure 6A). Then, using fresh skin explants extracted of a male mouse, we also observed a quick decrease on CPDs' levels from 20 to 60 min post‐sUV (Figure 6B). The agreement between these results indicates that our ex vivo skin explant model is suitable for analyzing not only DNA damage formation but also its repair. The observed rate of CPD repair in our skin explant model is faster than typically expected. However, the analysis of primary cells extracted from the same strain of mice confirms our findings and rules out technical artifacts such as issues with sample handling or DNA degradation, which could lead to incorrect interpretations of the data. This faster rate of repair may be due to e.g., an increased repair efficiency in our model or the specific conditions of our study, such as our optimized sUV exposure conditions.^12^

There is a need for a skin model that combines the natural physiology of skin while avoiding ethical issues associated with animal research. The skin explant model characterized and standardized in this work is most effective for short incubation periods of less than 6 days or can be frozen for at least 30 days and used. Our experimental results suggest that both DMEM and DMEM/F12 mixtures are suitable for longer incubation periods, but further research is needed to extend the viability of explants beyond 6 days. As demonstrated here, this model can be utilized to compare CPD damage and repair post‐sUV, for example, in response to topical dermatological treatments for photoprotection; it can be used to test the effectiveness of sunscreens and topical medications. Moreover, this model requires fewer mice than in vivo testing, reducing research costs by allowing multiple variables to be studied using skin from a single mouse. Freezing the tissue also extends the timeframe after euthanasia, providing researchers with greater flexibility in completing experiments. In conclusion, the ex vivo skin explant model effectively mimics the in vivo skin for up to 6 days in cell culture medium and at least 30 days when frozen, offering a broad range of potential applications that warrant further investigation.

## CONFLICT OF INTEREST STATEMENT

None of the authors have a conflict of interest to disclose.

## Data Availability

The data that support the findings of this study are available from the corresponding author upon reasonable request.

## References

[1] Jensen C , Teng Y . Is it time to start transitioning from 2D to 3D cell culture? Front Mol Biosci. 2020;7:33. 32211418 10.3389/fmolb.2020.00033 PMC7067892

[2] Muller I , Kulms D . A 3D organotypic melanoma spheroid skin model. J Vis Exp. 2018;135:57500. 10.3791/57500 PMC6101273 29863656

[3] Lebonvallet N , Jeanmaire C , Danoux L , Sibille P , Pauly G , Misery L . The evolution and use of skin explants: potential and limitations for dermatological research. Eur J Dermatol. 2010;20(6):671‐684. 20822970 10.1684/ejd.2010.1054

[4] Steinstraesser L , Rittig A , Gevers K , et al. A human full‐skin culture system for interventional studies. Eplasty. 2009;9:e5. 19198642 PMC2627306

[5] Peramo A , Feinberg SE , Marcelo CL . A putative in vitro organotypic model of molting with human skin explants. Arch Dermatol Res. 2012;304(2):145‐153. 22037627 10.1007/s00403-011-1187-z

[6] Lu Z , Hasse S , Bodo E , Rose C , Funk W , Paus R . Towards the development of a simplified long‐term organ culture method for human scalp skin and its appendages under serum‐free conditions. Exp Dermatol. 2007;16(1):37‐44. 17181635 10.1111/j.1600-0625.2006.00510.x

[7] Frade MA , Andrade TA , Aguiar AF , et al. Prolonged viability of human organotypic skin explant in culture method (hOSEC). An Bras Dermatol. 2015;90(3):347‐350. 26131864 10.1590/abd1806-4841.20153645 PMC4516099

[8] Keaven EP , Cox AJ . Organ culture of human skin From the Departments of Dermatology and Pathology, Stanford University School of Medicine, Palo Alto, California. J Invest Dermatol. 1965;44(3):151‐156. 14275691

[9] Neil JE , Brown MB , Williams AC . Human skin explant model for the investigation of topical therapeutics. Sci Rep. 2020;10(1):21192. 33273665 10.1038/s41598-020-78292-4 PMC7712775

[10] Vostalova J , Cukr M , Zálešák B , Lichnovská R , Ulrichová J , Rajnochová Svobodová A . Comparison of various methods to analyse toxic effects in human skin explants: rediscovery of TTC assay. J Photochem Photobiol B. 2018;178:530‐536. 29247925 10.1016/j.jphotobiol.2017.12.011

[11] Zhao H , Wu S . The effect of endothelial cells on UVB‐induced DNA damage and transformation of keratinocytes in 3D Polycaprolactone scaffold Co‐culture system. Photochem Photobiol. 2019;95(1):338‐344. 30160308 10.1111/php.13006 PMC6347483

[12] Bahamondes Lorca VA , McCulloch MK , Ávalos‐Ovando Ó , Govorov AO , Rahman F , Wu S . Characterization of UVB and UVA‐340 lamps and determination of their effects on ER stress and DNA damage. Photochem Photobiol. 2022;98(5):1140‐1148. 34932214 10.1111/php.13585 PMC9209597

[13] Walmsley GG , Maan ZN , Hu MS , et al. Murine dermal fibroblast isolation by FACS. J Vis Exp. 2016;107:53430. 10.3791/53430 PMC4781205 26780559

[14] Vigelso A , Dybboe R , Hansen CN , Dela F , Helge JW , Guadalupe Grau A . GAPDH and beta‐actin protein decreases with aging, making stain‐free technology a superior loading control in Western blotting of human skeletal muscle. J Appl Physiol (1985). 2015;118(3):386‐394. 25429098 10.1152/japplphysiol.00840.2014

[15] Xu Q , Zhu L , Wang G , et al. Application of cryopreserved autologous skin replantation in the treatment of degloving injury of limbs. J Plast Reconstr Aesthet Surg. 2022;75(7):2387‐2440. 10.1016/j.bjps.2022.04.006 35508521

